# Evaluation of FKBP5 as a cortisol activity biomarker in patients with ACTH-dependent Cushing syndrome

**DOI:** 10.1016/j.jcte.2021.100256

**Published:** 2021-06-06

**Authors:** Irina Bancos, Betul Ayse Hatipoglu, Kevin C.J. Yuen, Lakshmi Chandramohan, Sandeep Chaudhari, Andreas G. Moraitis

**Affiliations:** a Division of Endocrinology, Mayo Clinic, 200 First Street SW, Rochester, MN 55905, USA; b Endocrinology and Metabolism Institute, Cleveland Clinic, 9500 Euclid Avenue, F20, Cleveland, OH 44195, USA; c Swedish Neuroscience Institute, Swedish Pituitary Center, 550 17th Avenue, Suite 400, Seattle, WA 98122, USA; d NeoGenomics Laboratories, Inc., 7256 South Sam Houston Parkway West, Suite 300, Houston, TX 77085, USA; e Advance Research Associates, 2350 Mission College Blvd #825, Santa Clara, CA 95054, USA; f Corcept Therapeutics, 149 Commonwealth Drive, Menlo Park, CA 94025, USA

**Keywords:** FKBP5, Biomarker, Cushing syndrome, Cortisol activity

## Abstract

**Purpose:**

To evaluate the performance of FKBP5 as a cortisol activity biomarker in patients with ACTH-dependent Cushing syndrome (CS).

**Methods:**

This was a prospective, multicenter, nonrandomized, noninterventional study of a cortisol activity biomarker in adult patients (≥18 years) with documented ACTH-dependent, endogenous CS. The impact of surgery on FKBP5 mRNA expression levels in these patients and the difference in expression levels between these patients and healthy controls were evaluated. Cortisol and biomarker samples were collected before and immediately after surgery. A custom NanoString assay was used to quantify FKBP5 mRNA expression levels. The same method was used to analyze healthy volunteer samples collected from a different study.

**Results:**

Surgery was considered successful in 14/24 patients (58.3%) and changes from baseline in serum cortisol were −92.6% (*P* = 0.0005) and −43.8% (not significant) in patients with successful and unsuccessful surgeries, respectively. A strong positive correlation between FKBP5 and cortisol levels was observed (before surgery: r = 0.72, *P* = 0.0002; after surgery: r = 0.85, *P* < 0.0001). After successful surgery, FKBP5 expression levels were similar to those of healthy subjects. In patients without surgical success, FKBP5 levels remained unchanged from baseline and distinct from healthy subjects (*P* = 0.0025).

**Conclusions:**

Our findings confirm that FKBP5 levels are higher in the presence of excess cortisol exposure in patients with CS and decrease to normal baseline levels after successful surgery. These findings suggest that FKBP5 can serve as a measure of biological cortisol activity and set the stage for the development of an FKBP5 mRNA expression assay as a biomarker of cortisol activity.

## Introduction

Cortisol, a glucocorticoid hormone secreted by the adrenal glands, has wide-ranging functions in the human body, including mediating the stress response and regulating metabolism, inflammatory response, and immune function [1]. Cortisol acts by binding to and activating glucocorticoid receptors (GR) expressed in almost every tissue of the body [2], [3]. In the absence of cortisol, the GR is maintained in a transcriptionally inactive form as part of a large multi-protein complex in the cytosol, but when bound to cortisol, the associated proteins dissociate [1]. The cortisol-receptor complex then translocates into the nucleus, where it exerts its physiological function by up- or down-regulating gene expression.

Chronic exposure to excess cortisol levels (Cushing syndrome [CS], hypercortisolism) is associated with increased mortality and substantial morbidity [4], [5], [6], [7], [8]. Endogenous CS is caused by adrenocorticotropic hormone (ACTH)-secreting pituitary or ectopic tumors or by cortisol-secreting adrenal tumors or adrenal hyperplasia. Currently, the diagnosis of CS relies on performing at least two of the following biochemical tests: 24-hour urine free cortisol (UFC), late-night salivary cortisol (LNSC), and overnight 1-mg or two-day low-dose dexamethasone suppression test (DST). These tests vary widely in their accuracy, normal ranges depend on the methodology used, and results must be interpreted in the context of the suggested cutoff values for each test [9]. In addition, some of these tests are also used to determine the response to medical or surgical therapy and to guide dose adjustments while on medical therapy.

However, cortisol levels do not accurately reflect cortisol activity or disease severity, which can create significant diagnostic and management challenges for physicians. Guarnotta et al. demonstrated that the degree of urinary hypercortisolism does not appear to correlate with the severity of CS and that the clinical phenotype and associated comorbidities are more appropriate in assessing disease severity [10]. The lack of association between cortisol levels and disease severity may in part be due to GR polymorphisms that either increase or decrease sensitivity to cortisol or other downstream GR abnormalities (e.g., GR complex transportation or recycling). A test that measures cortisol activity at the GR rather than circulating cortisol levels could yield more clinically meaningful results.

**Fig. 1:**
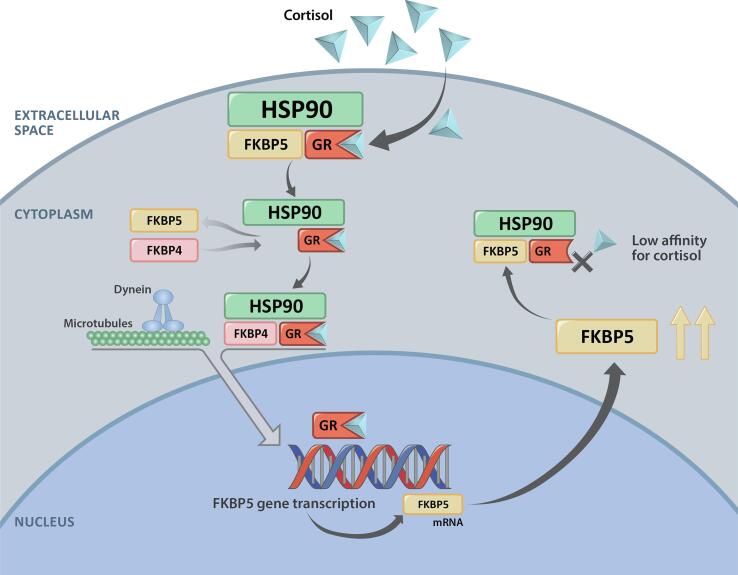
Illustration of FKBP5 mRNA expression. FKBP5, FK506 binding protein 51; FKBP4, FK506-binding protein 52; HSP90, heat shock protein 90; GR, glucocorticoid receptor.

FKBP5 (FK506-binding protein 51) has been proposed as one such potential cortisol activity biomarker, as preclinical studies in multiple species have shown that the induction of FKBP5 mRNA is directly regulated by GR agonism [11]. FKBP5 is a co-chaperone of heat-shock protein 90 (Hsp90) that regulates GR activity by reducing cortisol binding affinity [12] and restricting nuclear translocation [13]. FKBP5 interacts with the GR to maintain it in an unbound, inactive state within the cytoplasm, thus delaying translocation of the GR-cortisol complex into the nucleus (Fig. 1) [11]. FKBP4 (FK506-binding protein 52) binds to the cortisol-receptor complex only when FKBP5 dissociates, allowing the ligand-activated GR complex to translocate into the nucleus and exert its action as a transcription factor. The action of the ligand-activated GR complex on glucocorticoid response elements can then increase the amount of FKBP5 mRNA and ultimately increase FKBP5 protein production. In the presence of glucocorticoids, FKBP5 expression at both the mRNA and protein level is increased as part of an intracellular negative feedback loop [11]. A previous study in healthy volunteers [11] demonstrated that FKBP5 expression is sensitive to exogenous glucocorticoids. In that study, a time-dependent increase in FKBP5 mRNA expression was observed after oral administration of the synthetic glucocorticoid prednisone, and FKBP5 levels returned to baseline within 24 h of prednisone discontinuation. Whether a similar correlation exists between endogenous glucocorticoid levels, especially in patients with CS, and FKBP5 expression has not been previously evaluated.

The current study aimed to evaluate the performance of FKBP5 as a cortisol activity biomarker in patients with ACTH-dependent CS. The impact of successful surgery (i.e., resulting in biochemical remission) on FKBP5 mRNA expression levels was evaluated and expression levels in participants with CS and healthy controls were compared. The hypothesis tested in this study is that FKBP5 mRNA expression is increased in participants with CS and will decrease to normal after successful surgery. This study presents an initial step in developing FKBP5 as a biomarker of cortisol activity that could have potential application in the monitoring of patients with CS treated with GR antagonists.

## Subjects and methods

This study (NCT02922257) was a prospective, multicenter, nonrandomized, noninterventional biomarker specimen collection study in eligible adult patients (≥18 years) with documented ACTH-dependent, endogenous CS who were scheduled for pituitary or ectopic tumor surgery. The following were exclusion criteria: the planned use of preoperative and/or intra-operative glucocorticoids; the use of medical therapies for the treatment of CS (eg, cortisol synthesis inhibitors, somatostatin analogs, neuromodulators, or mifepristone); the use of medications that could potentially stimulate the expression of FKBP5 (testosterone or other steroid hormone analogues, oral contraceptives, or hormonal replacement therapies [14], [15]); and patients with ACTH-independent (adrenal) CS, because of the possible use of perioperative glucocorticoids. The study’s primary endpoint was the change in FKBP5 mRNA expression levels from baseline to after surgery. Successful surgery was defined as documented adrenal insufficiency based on serum cortisol levels of less than 5 μg/dL, as per the Endocrine Society guideline recommendation [16].

### Sample collection

Serum cortisol (1.0 mL) and biomarker whole blood (2.5 mL) were drawn at the same time from patients at defined time points: pre-surgical visit (Day −14 to Day 1); day of surgery (Day 1); post-surgical inpatient (Days 1–5) prior to initiation of glucocorticoid replacement therapy; and outpatient follow-up (post-surgical visits up to 3 years or until relapse). Data collected during outpatient follow-up will not be presented here. Serum cortisol specimens were collected as a paired collection with FKBP5 biomarker samples in the morning when possible (pre-surgery, outpatient follow-up), or based on the local standard of care (i.e., every 6 h until nadir; post-surgery Days 1–5). Biomarker specimens were collected in PAXgene™ blood RNA tubes (PreAnalytix, Switzerland) and sent to NeoGenomics Laboratories Inc. (Houston, TX) for mRNA expression analysis using NanoString nCounter technology. To ensure sample integrity, PAXgene™ blood RNA tubes were used because they reduce RNA degradation. Samples were kept at −80 °C until processed [17]. For comparison, FKBP5 samples from 26 healthy volunteers (18–60 years) were obtained from a separate study [18] and analyzed according to the same NanoString analysis as those collected as part of our study. To minimize the effect of the circadian rhythm on plasma cortisol levels and FKBP5 expression, healthy-volunteer FKBP5 whole blood samples were collected between 11 am and 2 pm [18].

### NanoString and RNA isolation methodologies

Total RNA from PAXgene™ blood RNA tubes was extracted using a PAXgene™ blood RNA extraction kit (QIAGEN, Germantown, MD, Catalog: 762164) according to the manufacturer’s protocol. Quantification and quality control (QC) results for the extracted RNA samples were obtained through the NanoDrop 2000 (Thermo Fisher Scientific, Wilmington, DE) and 2100 Bioanalyzer Instrument (Agilent Technologies, Santa Clara, CA). For quantitative mRNA expression profiling, a 33-gene custom nCounter panel (NanoString Technologies, Inc., Seattle, WA) consisting of 24 previously published genes (designated as endogenous genes) and 9 housekeeping genes was designed. Housekeeping genes were selected from publicly available databases based on stability and detectable expression levels across the biomarkers of interest. Additionally, 6 positive and 8 negative control genes were included as assay controls in the same panel, totaling 47 genes analyzed. Custom-designed probes included a 100-bp region targeting mRNA with 2 sequence-specific fluorescent-barcoded probes for each gene target. All samples were tested using the custom nCounter panel at 200 ng of total RNA input and hybridized overnight at 65 °C according to the manufacturer’s instructions. Unhybridized probes were removed with automated purification performed on an nCounter Prep Station (NanoString Technologies Inc., Seattle, WA), and the remaining target-probe complexes were transferred and bound to an imaging surface (cartridge) as previously described [19]. The cartridges were then transferred to the nCounter Digital Analyzer (NanoString Technologies Inc., Seattle, WA) for data collection and scanned at 550 fields of view following the manufacturer’s instructions for counting the digital barcodes representing the number of transcripts. Results were analyzed using nSolver v3.0 Analysis Software (NanoString Technologies Inc., Seattle, WA) using the manufacturer-recommended settings for QC and data analysis for gene expression analysis. The quality of the data was assessed using 4 data QC parameters in the nSolver program (imaging, binding density, positive control linearity, and positive control limit of detection). Only those samples that passed data QC were further analyzed. A normalization factor was calculated by obtaining the geometric mean of all housekeeping genes for each sample and applied to the raw counts of the nCounter output data to eliminate variability that was unrelated to the samples. Normalized counts per target per sample were used for statistical analysis.

Prior to testing clinical samples, assay performance of this 33-gene custom nCounter panel was verified and demonstrated 100% concordance and high correlation between NanoString and real-time quantitative reverse transcription polymerase chain reaction (qRT-PCR) (data not shown).

### Statistical analysis

SAS version 9.4 was used to generate all statistical analyses. Categorical variables were summarized by counts and proportions; continuous variables were summarized by median and range. Summary statistics for FKBP5 levels were summarized in bar charts. To assess the differences in cortisol levels between visits within each surgical outcome group, Sign tests were used. When specifically comparing differences between baseline values and post-baseline surgical outcomes, a Friedman’s test was used with alpha adjusted to 0.025. When comparing differences between healthy controls and successful/unsuccessful surgical outcomes, a Kruskal-Wallis test was used to test for overall differences while post-hoc comparisons were carried out using the Dwass, Steel, Critchlow-Fligner method.

To analyze the NanoString data, NanoString gene expression data for each target were normalized to the housekeeping genes included in the panel in nSolver v3.0 and log2-transformed for further analysis for differential expression. Data from joint samples were analyzed in R using unpaired T-tests followed by Benjamini and Hochberg multiple hypothesis correction.

## Results

Twenty-four consented patients with ACTH-dependent CS were enrolled in this study, of which 22 underwent surgery and had at least one paired pre- and one post-surgical cortisol/FKBP5 biomarker sample collected (evaluable population). Except for one patient with ectopic disease, all participants had CS of pituitary origin (Cushing disease). Baseline characteristics are summarized in Table 1. After surgery, 12 patients (54.5%) in the evaluable population showed serum cortisol levels of less than 5 μg/dL (defined as successful surgeries), while cortisol levels remained above the threshold in the remaining 10 patients (45.5%; defined as unsuccessful surgeries).

**Table 1:** Baseline characteristics in the safety population.

	Successful surgery (n = 14)	Unsuccessful surgery(n = 10)	Total (n = 24)
Age (years) median (range)	40.5 (19.0, 61.0)	51.0 (30.0, 63.0)	43.0 (19.0, 63.0)
Female, n (%)	12 (85.7)	7 (70.0)	19 (79.2)
Etiology of Cushing syndrome, n (%)			
Ectopic ACTH secretion	0 (0.0)	1 (10.0)	1 (4.2)
Cushing disease	14 (100.0)	9 (90.0)	23 (95.8)

ACTH, adrenocorticotropic hormone; UFC, urinary free cortisol.

### Cortisol levels pre- and post-surgery

Among the 22 patients in our study with paired cortisol/FKBP5 biomarker samples pre- and post-surgery, median serum cortisol level after surgery was 1.1 µg/dL in those with successful surgery compared to 12.6 µg/dL in those with unsuccessful surgery (Table 2). This corresponds to a change from baseline of –92.6% (*P* = 0.0005, successful surgery) and −43.8% (*P* = 0.1797, unsuccessful surgery), respectively.

**Table 2:** Serum cortisol measurements in evaluable patients before and after surgery (prior to glucocorticoid replacement).

	Successful surgery (n = 12)	Unsuccessful surgery (n = 10)
Cortisol at baseline (µg/dL) median (range)	16.5 (12.0, 50.2)	16.0 (7.1, 51.0)
Cortisol after surgery (µg/dL) median (range)	1.1 (0.8, 4.2)	12.6 (5.6, 22.4)
% Change from baseline median (range)	−92.6 (−98.0, −78.8)	−43.8 (−72.4, 215.5)
*P-*value*	0.0005	0.1797

* Sign test.

### FKBP5 mRNA expression levels pre- and post-surgery

Across the evaluable patients in this study, a strong positive correlation between FKBP5 and cortisol levels, both before and after surgery, was observed (before surgery: r = 0.72, *P* = 0.0002; after surgery: r = 0.85, *P* < 0.0001).

**Fig. 2:**
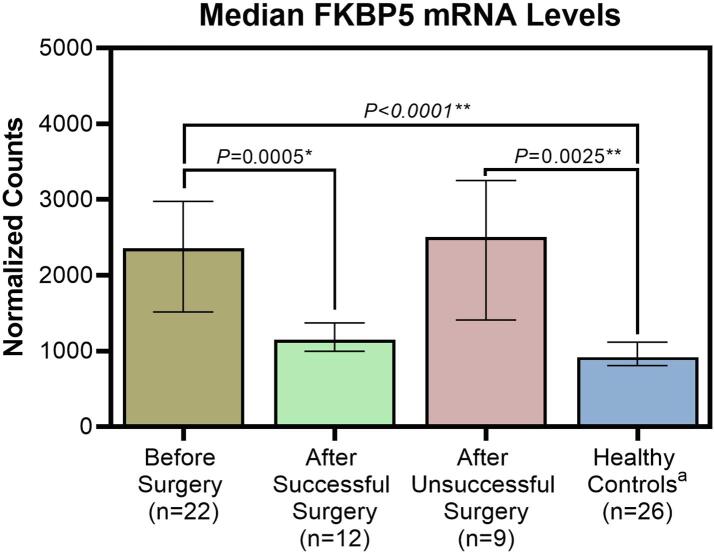
Median FKBP5 mRNA levels before/after surgery and in healthy controls. Data shown are median with first and third quartile. Differences between groups not statistically significant unless *P*-values are shown.* Friedman’s test, α = 0.025; ^**^ Dwass, Steel, Critchlow-Flinger method; Kruskal-Wallis *P* ≤ 0.0001. ^a^ Healthy control samples from Lee et al. [18].

Before surgery, median normalized FKBP5 mRNA counts were 2,357.3 ± 1,460.3 (median ± interquartile range) in patients with CS, compared to 918.7 ± 311.1 in healthy controls (*P* < 0.0001). Post-surgery, FKBP5 mRNA expression levels in study participants with successful surgical outcomes were not significantly different from those of healthy subjects (1,148.7 ± 372.8 vs 918.7 ± 311.1, *P* = 0.1843), while in patients with failed surgery, FKBP5 levels remained elevated (2,505.3 ± 1,843.4), unchanged from baseline (*P* = 0.7389), and distinct from healthy subjects (*P* = 0.0025) (Table 3 and Fig. 2).

**Table 3:** FKBP5 measurements in evaluable patients and healthy controls before and after surgery (prior to glucocorticoid replacement).

	FKBP5	*P-*value	
	Normalized counts	vs Healthy controls**	vs Before surgery***
Before surgery, median (range)	2357.325, (1071.43, 17215.80)	< 0.0001	—
After successful surgery, median (range)	1148.745, (725.71, 1732.09)	0.1843	0.0005
After unsuccessful surgery, median (range)	2505.250, (972.64, 5418.39)	0.0025	0.7389
Healthy controls*, median (range)	918.715, (661.68, 1735.91)	—	—

* Healthy volunteer samples from [18] collected between 11 am and 2 pm.

** Dwass, Steel, Critchlow-Flinger method, Kruskal-Wallis *P* ≤ 0.0001.

*** Friedman’s test, α = 0.025.

## Discussion

FKBP5 is a 51-kDa member of the immunophilin protein family that is involved in immunoregulation and the regulation of protein folding and trafficking. Notably, FKBP5 plays a key role in the regulation of steroid hormone receptors as part of the Hsp90 steroid receptor complex [20]. FKBP5 expression at both the mRNA and protein level is induced by glucocorticoids as part of an intracellular negative feedback loop for regulating GR agonism (Fig. 1). In healthy volunteers, Bali et al. found that treatment with a single dose of GR antagonist (mifepristone [Korlym®] or relacorilant; both Corcept Therapeutics, Menlo Park, CA) blunted prednisone-induced activation of the GR and resulted in a reduction of FKBP5 mRNA expression [11]. Bali et al. concluded that FKBP5 mRNA expression was a promising candidate for a biomarker of glucocorticoid activity since the induction of FKBP5 mRNA is directly regulated by GR agonism in multiple species.

Our study reports the first measurements of FKBP5 mRNA in patients with endogenous CS before and after surgery, and we have demonstrated a clear separation between successful and unsuccessful surgeries based on the post-surgery cortisol cutoff level recommended by the Endocrine Society [16]. Along with elevated serum cortisol levels (Table 2), patients with ACTH-dependent CS also showed significantly higher FKBP5 mRNA expression levels prior to surgery compared to healthy controls (Table 3). These findings confirm and expand upon previous studies, in which FKBP5 levels increased in response to glucocorticoid administration in vitro, ex vivo, as well as in rodents and healthy volunteers [11], [18], [21]. Additionally, our study found that after successful surgery, FKBP5 levels decreased to levels similar to those found in healthy volunteers (Table 3 and Fig. 2). In contrast, in patients who did not meet the criteria for surgical success, FKBP5 levels remained elevated similar to pre-surgery levels. These findings, although limited to ACTH-dependent causes of CS, support our hypothesis that FKBP5 can serve as a measure of cortisol biological activity and set the stage for the development of FKBP5 mRNA expression as a potential biomarker of cortisol activity in patients with CS.

The limitations of the current study include a relatively small sample size and that the healthy volunteer samples were not collected as part of the same study. Of the 75 healthy volunteers reported in Lee et al. [18], samples of 26 subjects were made available for use in the current study. Due to potential interactions with other steroid hormones, patients taking any steroidal hormones were excluded from the study. Testosterone and estradiol levels were not measured, and sex-related differences in expression levels of FKBP5 were not evaluated because of the small sample size. While we are not aware of the average age or male-to-female ratio among the 26 subjects, Lee et al. reported that FKBP5 expression levels did not differ significantly by sex or age. In our study, blood samples for cortisol and FKBP5 expression were collected in the morning when possible, while the FKBP5 expression samples of the healthy volunteers were collected between 11 am and 2 pm. However, the healthy volunteer samples went through the same process for analysis as those collected as part of our study.

The data presented here support the fact that FKBP5 mRNA expression is correlated with cortisol levels and elevated in patients with CS. FKBP5 mRNA expression returns to normal baseline values upon successful surgical resolution of the hypercortisolism. While surgical resection of the underlying tumor is usually the first-line treatment for patients with endogenous CS [22], [23], after failed surgery, tumor recurrence, or in patients not eligible for surgery, pharmacological reduction of cortisol activity can also be achieved with GR antagonists. In the setting of treatment with GR antagonists, measurement of cortisol levels in the urine, saliva, or blood using the current tests for hypercortisolism cannot be used to monitor therapeutic response. Instead, therapeutic assessment in these patients is based solely on changes in signs and symptoms over time [24], [25]. Directly measuring cortisol activity at the cellular level through FKBP5 mRNA expression could serve as a biomarker for the monitoring of these patients and is highly desired in clinical practice.

Additional studies directly investigating the utility of FKBP5 mRNA expression in patients with endogenous CS treated with GR antagonists could also prove very valuable. If validated, an FKBP5 mRNA assay could supplement or, in some cases, replace current diagnostic methods, such as the UFC, LNSC, or DST and could potentially supplement the current method of evaluating therapeutic efficacy based on the resolution of CS signs and symptoms. FKBP5 is currently being studied as a potential biomarker for cortisol activity in 2 ongoing phase 3 studies of the investigational selective GR modulator relacorilant (Corcept Therapeutics, Menlo Park, CA) in patients with endogenous CS (NCT03697109, NCT04308590).

## Funding

This study was funded by Corcept Therapeutics. Corcept Therapeutics was involved in the study design; collection, analysis, and interpretation of data; writing and reviewing the manuscript; and in the decision to submit the article for publication.

## Data availability

Some or all datasets generated during and/or analyzed during the current study are not publicly available but are available from the corresponding author on reasonable request.

## CRediT authorship contribution statement

**Irina Bancos:** Investigation, Writing - review & editing. **Betul Ayse Hatipoglu:** Investigation, Writing - review & editing. **Kevin C.J. Yuen:** Investigation, Writing - review & editing. **Lakshmi Chandramohan:** Methodology, Validation, Formal analysis, Writing - review & editing. **Sandeep Chaudhari:** Data curation, Software, Formal analysis, Visualization, Visualization, Writing - review & editing. **Andreas G. Moraitis:** Conceptualization, Methodology, Writing - original draft, Supervision.

## Declaration of Competing Interest

**Irina Bancos**: Consultant/Advisor - Corcept Therapeutics, CinCor, HRA Pharma, Strongbridge, Sparrow Pharmaceutics; **Betul Ayse Hatipoglu**: Employee - Cleveland Clinic; **Kevin C. J. Yuen**: Consultant/Advisor - Recordati, Sandoz, Novo Nordisk, Ipsen, Chiasma, Crinetics; Investigator/Researcher - Corcept Therapeutics, Novartis, Crinetics, Millendo, Ionis; **Lakshmi Chandramohan:** Employee - NeoGenomics Laboratories, Inc.; **Sandeep Chaudhari:** Employee - Advance Research Associates; Consultant/Advisor - Advance Research Associates, Corcept Therapeutics; **Andreas G. Moraitis**: Employee - Corcept Therapeutics.
